# Candida Spondylitis Considered as Microbial Substitution After Multiple Surgeries for Postoperative Lumbar Spine Infection

**DOI:** 10.7759/cureus.14995

**Published:** 2021-05-12

**Authors:** Yusuke Eda, Toru Funayama, Masaki Tatsumura, Masao Koda, Masashi Yamazaki

**Affiliations:** 1 Department of Orthopaedic Surgery, Faculty of Medicine, University of Tsukuba, Tsukuba, JPN; 2 Department of Orthopaedic Surgery and Sports Medicine, Tsukuba University Hospital Mito Clinical Education and Training Center, Mito, JPN

**Keywords:** candida, spondylitis, fluconazole, fungal disease

## Abstract

*Candida *spondylitis is a relatively rare disease. The primary risk factor is an immunocompromised status. Here, we report an immunocompetent patient who developed *Candida* spondylitis. The patient was a 70-year-old male. After multiple surgeries, he developed a fever and was diagnosed with chronic pyogenic spondylitis of the lumbar spine, which was treated by long-term antimicrobial therapy. However, his back pain persisted and the inflammatory response was prolonged. We performed posterior thoracolumbar pelvic fixation with a percutaneous pedicle screw system to stabilize the infected vertebral bodies and simultaneously performed a full-endoscopic intervertebral disc biopsy to identify the causative organisms. *Candida parapsilosis* was identified from a fungal culture of the biopsy specimen. The patient was diagnosed with *Candida *spondylitis and started on antifungal treatment with fluconazole. His back pain disappeared quickly after surgery, and up to the time of this writing, the patient has continued to receive fluconazole. We attributed the development of *Candida* spondylitis to the patient’s long-term antibiotic treatment of a postoperative infection of the lumbar spine, which was associated with multiple back surgeries. Fungal spondylitis, including spondylitis caused by *Candida* spp., should be suspected in patients, even immunocompetent patients, with intractable postoperative spinal infections and pyogenic spondylitis due to microbial substitution. Long-term antimicrobial therapy without definitive identification of the causative organism of a postoperative infection of the lumbar spine that is associated with multiple surgeries can be a cause of *Candida* spondylitis. A biopsy is strongly recommended for the definitive diagnosis.

## Introduction

*Candida* spondylitis is a relatively rare disease, occurring in 0.5% to 1.6% of all types of spondylitis cases. *Candida* spondylitis is strongly associated with immunocompromise due to conditions such as steroid therapy, neutropenia, and chronic granulomatous disease. In addition, other previously reported possible risk factors for *Candida* spondylitis are prior use of broad-spectrum antibiotics and central venous access devices. The incidence of *Candida* spondylitis is estimated to increase because of an aging population and improved diagnostic modalities and techniques [1,2].

Here we report an immunocompetent patient who developed *Candida* spondylitis after multiple surgeries for a postoperative infection of the lumbar spine.

## Case presentation

The patient was a 70-year-old male without a previous history of significant health problems, who underwent a partial lumbar laminectomy (L2/3 to L5/S1) for lumbar spinal stenosis at another hospital about nine years previously. After the initial surgery, a postoperative wound infection developed. Another wound infection occurred after a posterior lumbar interbody fusion (L3/4, L4/5) approximately six years previously. Six months after the second surgery, an anterior lumbar interbody fusion that consisted of a combination of a plate and screw fixation with autologous iliac bone grafting was performed for infectious pseudoarthrosis at L3/4. The patient developed persistent back pain postoperatively. An intraoperative specimen that was cultured after the third surgery was negative for bacteria and the presence of tuberculosis or a fungus was also ruled out by a histopathological examination of the specimen. Two years after the third surgery, all pedicle screws of posterior lumbar interbody fusion of second surgery were removed from the infected posterior implant. Six months before his present admission, the patient developed a fever and was diagnosed with chronic pyogenic spondylitis of the lumbar spine. He was treated with intravenous cefazolin (3 g/day) and clindamycin (1,200 mg/day) followed by an oral course of cefaclor (750 mg/day) and clindamycin (450 mg/day), followed by an oral course of minocycline (100 mg/day). Antimicrobial treatment was administered for a total of six months. The inflammatory response was attenuated temporarily, but resumed.

**Figure 1:**
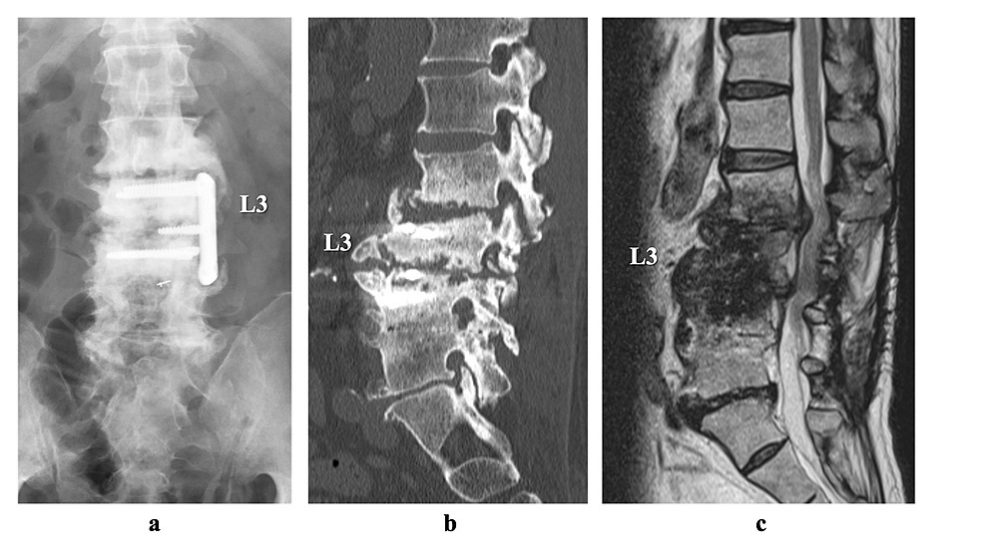
Images on admission. (a) Lumbar anterior-posterior plain radiograph of the lumbar spine showing the damaged L3/4 plate and L4 screw. (b) Computed tomography scan (sagittal slice) showing bone destruction of the vertebral endplates and osteosclerosis of the L2-4 vertebral bodies. (c) Sagittal T2-weighed magnetic resonance image showing the low-intensity signal of the L2-4 vertebral bodies.

For 9 years, he was referred to our hospital. He had no neurological findings, but the patient’s daily activities were strongly restricted because of persistent back pain. Upon admission, the patient was afebrile, and a peripheral blood sample revealed a mildly increased inflammatory response (white blood cells count 7100/µL, C-reactive protein [CRP] 1.02 mg/dL, and erythrocyte sedimentation rate [ESR] of 36 mm/h). Computed tomography (CT) showed bone destruction of the vertebral endplates and osteosclerosis of the L2-L4 vertebral bodies (Figure 1).

**Figure 2:**
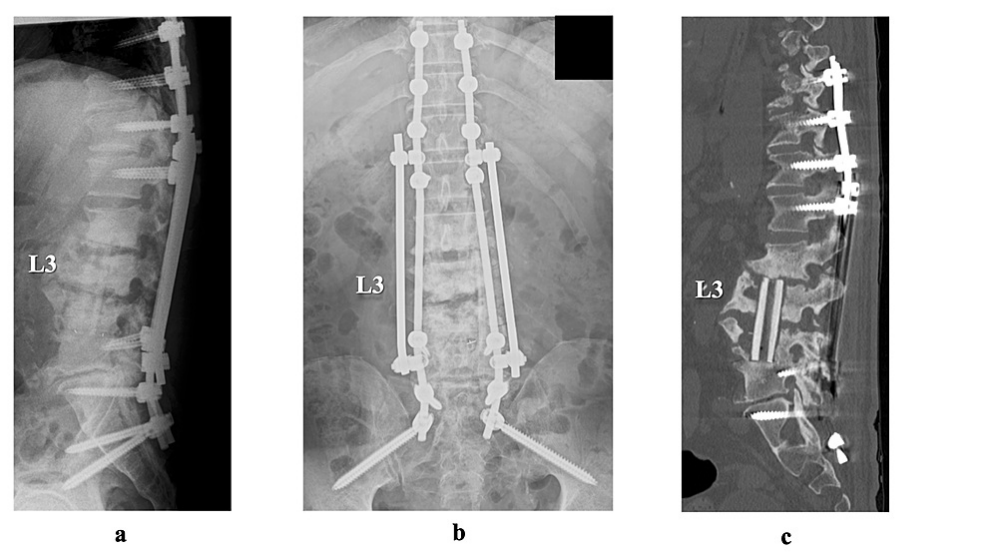
Post-operative images. (a, b, c) Posterior thoracolumbar pelvic fixation with percutaneous pedicle screw system to stabilize the infected unstable vertebral bodies, anterior plate removal, lesion curettage, and fibula graft via retroperitoneal approach.

We used a percutaneous pedicle screw system to perform a posterior thoracolumbar pelvic fixation, skipped the infected L2-4 vertebral bodies (Th10 - sacral-alar-iliac), in order to stabilize the infected unstable vertebral bodies, and simultaneously performed a full-endoscopic biopsy of the intervertebral disc of L3/4 to identify the causative organisms. *Candida* parapsilosis was identified from a fungal culture of the biopsy specimen of the intervertebral disc of L3/4. Altogether, the patient’s findings were diagnosed as *Candida* spondylitis, and antifungal treatment was started consisting of intravenous fluconazole (800 mg/day) three weeks, followed by an oral administration of fluconazole (400 mg/day). Two weeks after the posterior stabilization and biopsy, we removed the anterior plate and screws and performed curettage of the lesion and a fibula graft via a retroperitoneal approach (Figure 2). The patient’s back pain rapidly resolved after the procedure. At the time of this writing, he has continued to take fluconazole.

## Discussion

The following magnetic resonance imaging (MRI) findings have been reported to be characteristic of *Candida* spondylitis: change in the signal and destruction of the vertebral endplate in two adjacent vertebral bodies, a small paraspinal abscess, and low-intensity signal on T2-weighted imaging. By contrast, pyogenic spondylitis and tuberculous spondylitis generally show high-intensity signal on T2-weighted imaging [3]. Because both the characteristic MRI and CT findings are not specific for *Candida* spondylitis, to differentiate pyogenic spondylitis from *Candida* spondylitis using imaging findings only is difficult [4].

Guidelines for the diagnosis of pyogenic spondylitis include obtaining two sets of blood cultures for both aerobic and anaerobic bacteria, and obtaining the ESR and the CRP level. Fungal blood cultures are also recommended for patients at risk of fungal infection, such as those with immunocompromised conditions [5].

*Candida* spondylitis usually progresses more slowly and has fewer symptoms than pyogenic spondylitis. Patients with *Candida* spondylitis complain of mild back pain and lack signs/symptoms of fever or sepsis. The paucity of clinical signs/symptoms can lead to a delayed diagnosis. Therefore, an adequate biopsy specimen is essential for diagnosis [2,4]. In our patient, a biopsy obtained during a full endoscopic lumbar discectomy was needed for the definitive diagnosis.

Previously, there have been several reports of *Candida* spondylitis in immunocompetent patients. However, most of these reports were in patients with risk factors such as hepatitis C and prior use of central venous catheterization. There were only four reports of *Candida* spondylitis in immunocompetent patients without risk factors (Table 1). It took three weeks to four months to make the diagnosis. In all the reports, Candida could be detected not by simple blood culture but by biopsy or intraoperative specimens. Three to six months of antifungal therapy was needed to obtain the cure of *Candida* spondylitis. Together with the previous cases, the current case suggests that identification of the fungal species by the collection of local specimens is important for the diagnosis of *Candida* spondylitis and that a longer duration of antifungal therapy was unavoidable until cure.

**Table 1:** Reports of Candida spondylitis on immunocompetent patient.

References	Age	Segments	Spinal intervention	Duration to diagnosis	Cultures	Surgical treatment	Duration of antifungal treatment	Prognosis
Torres-Ramos et al. 2004 [6]	69; female	T8-9	None	4 months	Intraoperative cultures	Debridement Fusion	3 months	cured
Cho et al. 2010 [7]	70; female	L5-S1	Lumbar discectomy on L5-S1	2 months	Intraoperative cultures	Debridement	4 months	cured
Werner et al. 2011 [8]	40; female	L3-4	Lumbar discectomy on L5-S1, lumbar epidural steroid injection, lumbar facet injection, lumbar discogram	3 weeks	Intraoperative cultures	Debridement	6 months	cured
Darrieutort-Laffite et al. 2013 [9]	22; female	L1-2	None	4 months	Biopsy	None	6 months	cured
Current case	70; male	L2-4	Multiple spine surgeries	6 months	Full endoscopic discectomy	Debridement fusion	9 months	cured

Our patient did not have any risk factors for *Candida* spondylitis such as diabetes mellitus, or the use of steroids, prior use of broad-spectrum antibiotics, or a central venous catheter. Our patient’s *Candida* spondylitis might have been associated with microbial substitution by his long-term antibiotic therapy. There are several possibilities of the origin of candida infection. Both surgical contamination and hematogenous infection originated from the oral cavity are the most likely candidates. We are planning to terminate fluconazole treatment based on the imaging findings of bony union.

## Conclusions

Fungal spondylitis due to *Candida* spp. can develop in association with antibiotic therapy and should be considered for patients, including immunocompetent patients, with intractable postoperative spinal infections and pyogenic spondylitis. Multiple surgeries and long-term antimicrobial therapy without definitive microbial identification of a biopsy specimen can be associated with *Candida* spondylitis. Therefore, a biopsy is strongly recommended for the definitive diagnosis.
